# Making meaning of trauma in psychosis

**DOI:** 10.3389/fpsyt.2023.1272683

**Published:** 2023-11-03

**Authors:** Nienke van Sambeek, Gaston Franssen, Stefan van Geelen, Floortje Scheepers

**Affiliations:** ^1^Department of Psychiatry, University Medical Center Utrecht, Utrecht, Netherlands; ^2^Faculty of Humanities, University of Amsterdam, Amsterdam, Netherlands; ^3^Education Center, University Medical Center, Utrecht, Netherlands

**Keywords:** trauma, psychosis, meaning-making, narrative identity, service-user perspective, personal recovery, stigma, narrative analysis

## Abstract

**Background:**

Finding new meaning and identity in the aftermath of trauma has been identified as a key process of mental health recovery. However, research indicates that this meaning-making process is compromised in people with psychosis. Considering the high prevalence, yet under-treatment of trauma in people with psychosis, it is urgent to gain insight into how their meaning-making process can be supported.

**Aim:**

To gain insight into how people with psychosis make meaning of trauma and identify barriers and facilitators in their meaning-making process.

**Methods:**

Qualitative inquiry of *N* = 21 interviews transcripts from the Dutch Psychiatry Storybank. We included interviews of people who (a) lived through multiple psychotic episodes, and (b) spontaneously addressed traumatic experiences in a low-structured interview. Storyline analysis was performed to gain insight into the meaning-making of trauma within their self-stories. Psychosocial conceptualizations of narrative identity were used to inform the analysis. A data-validation session with four experts-by-experience was organized to check and improve the quality of our analysis.

**Results:**

We identified four different story types: (1) Psychiatry as the wrong setting to find meaning; (2) The ongoing struggle to get trauma-therapy; (3) Exposure to trauma as a threat to a stable life, and (4) Disclosure as the key to resolving alienation. Each story type comprises a different plot, meaning of trauma withing the self-story, (lack of) integration and barriers and facilitators in the meaning-making process. Overall, barriers in the meaning-making process were mostly situated within mental healthcare and stigma-related. People felt particularly hindered by pessimistic ideas on their capacity to develop self-insight and cope with distress, resulting in limited treatment options. Their process of adaptive meaning-making often started with supportive, non-judgmental relationships with individuals or communities that offered them the safety to disclose trauma and motivated them to engage in a process of self-inquiry and growth.

**Conclusion:**

The outcomes illuminate the social context of the meaning-making challenges that people with psychosis face and illustrate the devastating influence of stigma. Our outcomes offer guidance to remove barriers to adaptive meaning-making in people with psychosis, and can help clinicians to attune to differences in the meaning-making of trauma.

## 1. Introduction

People diagnosed with psychotic disorders, such as schizophrenia (1),^1^ experience disproportionately high rates of past and ongoing trauma in their lives (2, 3). They are at higher risk of developing post-traumatic stress disorder (PTSD) than people in the general population (4), with the majority of studies reporting a 20 to 30% prevalence rate (5).

The association between trauma and psychosis is firmly established, yet their interplay is complex and subject to ongoing revision [see (6–9)]. To date, (childhood) trauma as a risk factor for developing psychosis has been best established in the available evidence (10, 11). Several studies have demonstrated that the experience of childhood trauma is predictive for the onset of psychosis in persons at clinical high risk (12). A meta-analysis by Varese et al. (13) suggested that if childhood adversity was eliminated, one-third of adult psychosis would not occur. Other pathways between trauma and psychosis are being increasingly studied. Morrison et al. (6) proposed an integrative model of the spectrum of trauma reactions, outlining three routes between trauma and psychosis: (1) trauma may lead to psychosis, (2) psychosis and related experiences can themselves give rise to PTSD, and (3) both psychosis and PTSD may lie on a spectrum of shared reactions to emotional trauma. There is emerging evidence for a psychotic subtype of PTSD (14) and for PTSD as a result of psychotic symptoms and involuntary treatment experiences (15, 16).

Unfortunately, trauma and PTSD in people with psychosis often remain under-detected in mental healthcare (17, 18). Research of de Bont et al. (19) indicates an estimated under-report of PTSD of as much as 96,9% in clinical practice. As a consequence, many people with psychosis are unlikely to receive appropriate treatment for post-traumatic stress (20). Such disregard of trauma has severe negative consequences, as post-traumatic stress in psychosis is associated with worse functioning, lower quality of life, and higher levels of positive symptoms, neurocognitive impairments and general psychopathology (5). Consequently, both clinicians and patient representatives advocate for better, trauma-informed care for people with psychosis (21–23). In order to design such care, it is important to gain insight into the perspectives of people with psychosis themselves.

Previous qualitative research inquiring these perspectives identified that finding new meaning and identity after disruptive experiences is a key aspect of the process of personal recovery from schizophrenia and other forms of severe mental illness (24–26). Moreover, a review on personal recovery in psychosis found that coming to terms with past stress and trauma was an important first stage in this process (27). The role of trauma in personal recovery has only recently gained more attention (28), and studies offering in-depth insight into the meaning-making of trauma are scarce. The few studies we found on this subject indicated that, although trauma and adversity are often part of the illness explanations of people with psychosis (29, 30), they experience a lack of opportunities to discuss and address trauma, both within and outside mental healthcare (31–33).

With the present study, we aim to provide deeper insight into how people with psychosis make meaning of trauma. Specifically, we are interested in narrative meaning-making of trauma in the context of the stories people create about their selves and lives. By telling stories, people connect life events into a meaningful whole, leading to a certain extent of narrative integration (34). In order to better understand how narrative meaning-making can be disturbed and enhanced in response to trauma and psychosis, we will use conceptualizations of narrative identity as a theoretical framework.

Narrative identity has been defined by McAdams as: “The internalized and evolving story of the self that a person constructs to make sense and meaning out of his or her life (…) and that serves to explain, for the self and others, how the person came to be and where his or her life may be going.” (35, p. 99). The development of such self-stories is theorized to be a developmental psychological process that requires several cognitive skills. Most importantly, it requires the meta-cognitive skill of autobiographical reasoning: the reflexive activity of creating relations between different parts of one’s past, present, and future life and drawing conclusions about one’s personality and development (36, 37). In his research, McAdams demonstrated that in constructing self-stories, people strongly draw on prevailing cultural norms, metaphors and themes that are present in the narratives they encounter in their social life (38). Accordingly, he defined narrative identity as a psychosocial construction: “Any persons’ particular narrative identity is a co-authored, psychosocial construction, a joint product of the person him/herself and the culture wherein the person acts, strives and narrates.” (35, p. 112). In this study, we will draw on this psychosocial understanding of narrative identity to study the meaning-making of trauma in people with psychosis. First, we will shortly discuss how narrative identity can be affected by the experience of trauma and psychosis, respectively.

In psychological guidelines and research, traumatic events are described as experiences that can challenge a persons’ view of the world as a just, safe, and predictable place (39) and disrupt previous taken for granted beliefs and expectations (40). In terms of narrative identity, trauma can shatter a persons’ assumptive world and lead to a disrupted self-story (41). Neimeyer and colleagues distinguish different forms of narrative disruption that can result from trauma: Disintegration of the self-narrative occurs when confrontation with traumatic events disrupt a person’s sense of continuity of self, as the person one becomes after such experiences can feel radically different from the person one used to be (42). Furthermore, narrative dissociation entails a process in which the traumatic memory is excluded from conscious memory, but also from the personal story shared in a social context, resulting in a “silent story” that blocks integration into the self-narrative (43, p. 133). Lastly, narrative dominance can disrupt people’s own attempts at meaning-making by imposing an external, non-preferred identity. People, for example, can experience that their individuality disappears behind the universal label of a psychiatric disorder (42, p. 169).

As much as self-narratives can become disrupted by traumatic experiences, then, they simultaneously have the potential to negotiate or resolve such disruptions or “breaches” (44). In fact, storytelling is theorized to be motivated by biographical disruptions such as trauma and illness, as it can give people the means to render these experiences and their impact comprehensible (45). Especially in low-control situations, such as traumatic events, meaning-making may be the most adaptive coping response (46). It requires the reconstruction of a new assumptive world, which accounts for the traumatic experiences, yet is psychologically more comforting (47) and that is not only viable to the person itself but also creates support from relevant others (41). Empirical studies confirm that people can grow from adverse experiences through processes of meaning-making, and that adaptive meaning-making is associated with various forms of wellbeing and growth in both the general population (48, 49) and in people with psychosis (50, 51).

We only found one study that specifically inquired narrative meaning-making of traumatic experiences by people with psychosis. In this study, the authors found that participants reported high levels of traumatic childhood experiences in a questionnaire, but rarely mentioned, let alone integrated them in their personal narratives (52). Difficulty integrating personal experiences might complicate meaning-making of trauma in people with psychosis. Based on a systematic review of narrative identity research, Cowan et al. (53) concluded that one of the main features of the narratives of people with psychosis spectrum disorders is a detached narration style, including incoherence and “absence of meaning.” That is, difficulties interpreting life events and connecting them to each other and the present self. Such narrative disruptions have often been linked to impairments in memory and meta-cognitive skills which are considered a core feature of psychosis spectrum disorders (54). Other authors have drawn attention to the social factors that might complicate narrative meaning-making in the psychosis spectrum. For example, according to Roe and Davidson (55), the very perception of people diagnosed with schizophrenia as lacking reason and insight has led to an approach in psychiatry in which they are abandoned to the illness, dismissing rather than inviting narrative. Instead of focusing on deficits, they advocate for focusing on people’s efforts to overcome the disruptions that are introduced by the illness and its consequences.

Previous research on meaning-making efforts has mainly focused on the integration of illness-experiences into the self-stories of people with psychosis. Decades ago McGlashan et al. (56) already related differences in recovery from schizophrenia to differences in integration of illness experiences into identity. They observed two opposing recovery styles: “integration” and “sealing over.” Integration was characterized by people’s awareness of continuity in their mental activity and personality from before, during and after the psychotic experiences. People displaying this style tended to be curious about their experiences and prone to elicit help of others to understand them. By contrast, people with a sealing over recovery style tended to isolate the psychotic experience, which they experienced as alien and interruptive. They were disinclined to investigate the psychotic experience and perceived it as separate from their personal problems. Similar patterns have been found in a more recent narrative study of the experiences of voice hearers by De Jager et al. (57), in which the authors distinguish between people with a “turning toward/empowerment” and a “turning away/hibernation” recovery style. Several studies suggest that a recovery style directed at turning toward and integrating experiences predicts better functioning on the long term and that meaning-making styles in psychosis are not static but can change over time (58–61). These findings support a more hopeful perspective on the possibility of people with psychosis to integrate disturbing experiences.

In this study we will work from a recovery-oriented perspective, assuming that adaptive meaning-making of traumatic experiences is a possibility for people with psychosis. Drawing on psychosocial conceptualizations of narrative identity, we understand adaptive meaning-making as a dynamic and dialogical process of integrating traumatic experiences into the self-story, in a way that makes them more bearable and elicits social support.

Our research questions were: (1) How do people with psychosis make meaning of traumatic experiences in the context of their self-stories? (2) What have they experienced as barriers and facilitators in their meaning-making process? (3) What differences in (lack of) integration of traumatic experiences into the self-story can be identified?

## 2. Materials and methods

### 2.1. Research design

We performed a qualitative, narrative study of stories from people with psychosis. A common practice in the study of narrative identity is to code and rate narratives with standardized instruments, thus quantifying them (48). However, for exploring new avenues and complexity, qualitative methods are more suitable (62). These methods allow for understanding how people construct and negotiate their identities, and take into account their biographical trajectories (63). Our study is situated within a interpretivist-constructivist research paradigm (64). We chose a narrative, holistic approach to data-analysis that is particularly suitable for mental health recovery research (65).

### 2.2. Setting, procedure, and data-collection

This study is part of the “Psychiatry Storybank” from the University Medical Center of Utrecht (Netherlands). In this ongoing, long-term project we collect stories of service-users through low-structured interviews. Our aim is to gain in-depth understanding of service-users’ perspectives on mental illness, care and recovery, in order to improve psychiatric services. The project was evaluated by The Medical Ethical Review Committee of the University Medical Center of Utrecht, who confirmed that the Dutch Medical Research Involving Human Subject Act (WMO) did not apply. Subsequently, official approval of this study by the Medical Ethical Review Committee was not required (reference number WAG/mb/16/030724).

Participants in the project were people that made use of psychiatric services in the Netherlands. Apart from acute crisis, no exclusion criteria were formulated. Participants were recruited through convenience sampling: People could sign up through our project website, advertised on (social) media and by word-of-mouth. Participation was voluntarily and people were informed about their possibility to withdraw from the study at any time. After signing up, participants were first contacted by phone to discuss the aim and process of participation. An information letter with detailed information about participation and privacy was sent afterward. When people gave their consent to participate, the interview was planned and conducted at a place of the participants choosing. During the COVID-19 pandemic, participants were offered the possibility to be interviewed online, through video-calling. All participants gave their written informed consent. The interviews were transcribed verbatim and stored anonymously in our secured database.

The interviews were conducted by junior mental-health professionals that were trained and supervised by the Psychiatry Storybank team, including the first author. Interviewers were trained to invite participants to share what matters to them and connect as much as possible to their evolving story, while further exploring and deepening this story using five topics [identity, social participation, connectedness, (psychiatric) help and future perspectives, see Supplementary Appendix I]. After first observing an interview, new interviewers received detailed and systematic feedback on their first two interviews (see Supplementary Appendix II). Two reflection sessions with the interviewers were organized to discuss and address common points of feedback. These included the need to “unlearn” structured interview techniques and create more space for sensitive subjects. More details are provided in van Sambeek et al. (66). Note that the current study was designed after data-collection and that interviewers were unaware of the focus on the subject of trauma.

### 2.3. Interview selection and participant characteristics

From the Psychiatry Storybank database, we purposefully selected interviews of (former) service-users with psychosis that spontaneously addressed traumatic experiences. Firstly, from a total of 103 service-users’ interviews, we selected interviews from participants that experienced multiple psychotic episodes or reported to be diagnosed with a psychotic disorder (*N* = 33). From a transdiagnostic view, we assumed that diagnostic differences would be subordinate to the shared experience of living with (the fear for) recurrent psychosis. Hence, we included participants with different diagnoses. The second step was to identify interviews in which trauma was addressed. In the literature, there is ongoing discussion on what “counts” as a traumatic event. The DSM-5 criterion to identify exposure to traumatic events (“actual or threatened death, serious injury or sexual violence”) (67) has been criticized for being defined from an outside perspective, while it is the subjective appraisal of the event that determines the individuals stress reaction (68). Additionally, it excludes the psychosis-related traumatic events that are commonly reported by patients (69). As we were interested in personal meaning-making, the subjective experience of the participants was leading in our identification of trauma. Based on a first reading, we included interviews in which the participant explicitly referred to having experienced trauma, with or without providing further details. This method resulted in the inclusion of 18 interviews. In order to maximize variation in meaning-making, we additionally included interviews in which the participants did not literary use the word “trauma” but referred to events that they had clearly experienced as overwhelming and shocking and as threatening their safety or the safety of loved ones (68).^2^ This method yielded inclusion of three additional interviews. The 21 included interviews were conducted between January 2019 and December 2022. A total of 14 of them were conducted face-to-face and seven interviews were conducted through video-calling. Interviews lasted 63 min on average. Characteristics of the participants and experienced trauma are summarized in Tables 1, 2.

**TABLE 1:** Characteristics of the participants (*n* = 21).

Sex	Participants (*n*)	Relational status	Participants (*n*)
Male	7	Single	12
Female	14	Partner/married	9
**Employment**		**Children**	
Employed	6	Yes	11
Unemployed	6	No	10
Voluntary work	9		
		**Migration background**	
		Yes	4
		No	17
**Highest education**		**Age (μ = 47)**	
Primary school	1	19–30	2
Secondary school	3	31–40	4
Vocational education	5	41–50	7
Professional education	7	51–60	7
University	5	>60	1
**Primary diagnosis**		**Number of hospitalizations (μ = 4)**	
Bipolar disorder	8	≥5	5
Schizophrenia	4	2–5	11
Schizoaffective disorder	4	1	5
Unspecified psychotic disorder	5		
**Currently using psychotropic medication?**		**Currently using psychiatric services?**	
Yes	17	Yes	18
No	4	No	3

**TABLE 2:** Type of traumatic experience disclosed in the interview (multiple entries).

Childhood	Participants (*n*)	Adult life	Participants (*n*)
Sexual abuse	6	Sexual abuse	2
Physical assault	1	Physical assault	2
Emotional abuse	2	Domestic violence	3
Neglect	2	Burglary	1
Bullying	3	Bullying/intimidation	3
Victim of car-accident	1	Victim of car-accident	1
		Life threatening birth complications	1
Loss or permanent separation of parent	4	Suicide of loved-one	2
Witnessing fatal accident of family member	1	Witnessing kidnapping of family member	1
Witnessing suicide attempt of parent	1		
Unspecified trauma	2	Unspecified trauma	1
		**Psychosis-related**	
		Illness experiences (e.g., threatening hallucinations or self-harming behavior).	5
		Treatment experiences (e.g., seclusion, coercion)	8

### 2.4. Data analysis

To analyze narrative meaning-making of trauma, we performed storyline analysis as proposed by Murray and Sools (70). Several important principles of a holistic approach are integrated within this method, such as analysis of the story as a whole unit, regard for both content and form, and attention for the context in which the story is produced (71). Storyline analysis consists of analyzing different storyline elements (setting, characters, events/acts, intention, and means) in coherence, to eventually identify the breach within the story. A breach refers to friction in the assumptive world of the narrator. An imbalance between two story elements is seen as an indicator of such breach. In the subsequent steps of analysis, the identified storylines are positioned within the interactional context of the interview and the broader social-cultural context. In the last step of analysis, individual storylines are compared in order to identify more general story patterns, or plots across cases. We adapted the guidelines for storyline analysis to our specific research questions, theoretical framework and data, after a first reading of all interviews. The steps of storyline analysis are designed to support bottom-up analysis, which starts with detailed analysis of the unique words and meanings created by participants, and gradually proceeds to move up and become more theory driven (70, p. 139). For the latter, we made use of the theoretical elaborations of narrative disruptions and narrative integration that are described in the introduction. To recognize different forms of (lack of) integration of trauma on a textual level, we made use of indicators from the narrative identity literature, such as thematic and causal coherence. Causal coherence refers to the extent to which the narrator connects past, present and future self, reflecting continuity of the self-experience. Thematic coherence refers to the extent to which the narrative is centered around an overarching theme, life lesson, value or principle and indicates a process of reinterpretation and reflection (72, 73). A detailed overview of all steps of analysis and the use of these concepts can be found in Supplementary Appendix III. Comparative analysis led to the identification of four story types with different plots and forms of (lack of) integration. These story types are not intended as static categorizations, but as context dependent and evolving narrative forms. In order to provide useful entrees for personalizing trauma-informed care, barriers and facilitator were analyzed of each story type separately.

### 2.5. Trustworthiness

We acknowledge that researchers’ perspectives inevitably influence what is “found” in analysis. For example, all members of the multidisciplinary research team are proponents of recovery-oriented care, and our study is explicitly designed to align this vision. From our point of view, subjectivity is not to be avoided but to be critically worked with: by engaging in constant reflection and dialog about researchers’ assumptions and interpretations, in combination with systematic analysis. We made use of multiple strategies from international guidelines for good practice in qualitative research (74). To enhance transparency and rigor, researchers’ assumptions, reflections and disagreements were recorded in a logbook, providing insight into adaptations in the research process and researchers’ motivations. For instance, discussion of critiques on the concept of narrative coherence as a measure of healthy meaning-making resulted in adaptations in analysis to show multiple facets of meaning-making. After extended familiarization with the data, we held several cross-reading sessions within the research team to compare and broaden our understanding of the narratives. Subsequently, each narrative was analyzed in depth by the first author. Story elements were systematically coded in ATLAS-ti software, and detailed memos and reports of analysis were written and discussed within the team to support the comparative analysis. In order to enhance the credibility and authenticity of our findings, we organized a data-validation session with experts-by-experience. Participants were recruited through the mailing list of “Anoiksis,” the Dutch association for people with psychosis. We invited members with firsthand experience of both trauma psychosis to reflect on our preliminary results. Seven people reacted to the mailing, of which three were present at the online meeting (150 min). One person was consulted individually by telephone upon request. The four consulted experts were two men and two women between 31 and 52 years old. Three of them had engaged in trauma-therapy. They helped to further refine our results and interpret the meaning-making patterns we identified. Additionally, three overarching feedback points were brought forward. Firstly, the experts raised important questions about the concept and process of meaning-making itself. This feedback was processed in this article by further specifying what adaptive meaning-making entails. Secondly, they pointed to the role of health-inequalities in getting professional help with meaning-making of trauma. Although most of them had eventually found satisfying help, they emphasized their privileged position in terms of resources (for example: being able to verbalize care needs and having the financial means to pay for unreimbursed trauma-care). Third, experts recognized elements from most story types and reported changes in meaning-making. For instance, most of them had previously believed that confronting trauma would lead to destabilization.

## 3. Results

We identified four different story types in our sample: (1) Psychiatry as the wrong setting to find meaning; (2) The ongoing struggle to get trauma-therapy; (3) Exposure to trauma as a threat to a stable life, and (4) Disclosure as the key to resolving alienation. An overview of the characteristics of these story types can be found in Tables 3–5. Below, we will give an in-depth description of each story type by first characterizing the plot and the meaning of trauma within the self-story (Table 3). Secondly, we will describe the barriers and facilitators that narrators have encountered in their process of meaning-making of trauma (Table 4). Finally, we will characterize the (lack of) integration of trauma storylines into the self-story (Table 5). Distinguishing characteristics of the narrators are given in the introduction.

**TABLE 3:** Plot and meaning of trauma within self-story.

Story type	Breach	Plot summary	Meaning of trauma within self-story	Relation to psychosis
Psychiatry as the wrong setting to find meaning	Setting does not support realization of intention.	The mental healthcare setting where the protagonist expects to gain insight, lacks the basic conditions to support meaning-making.	Trauma needs to be acknowledged as an explanation for a disrupted life.	Trauma as a cause and consequence of psychosis.
The ongoing struggle to get trauma-therapy	Hinderers are too powerful to realize intention.	Despite ongoing efforts, the protagonist remains excluded from the desired trauma therapy.	Trauma needs to be confronted and felt through in order to recover.	Psychosis as a way of coping with unprocessed trauma.
Exposure to trauma as a threat to a stable life	Intentions are shifting and seem incompatible.	The protagonist recently began to see new future perspectives but is insecure to take any risk that might trigger trauma and disturb stability.	Trauma needs to be avoided, for it disrupts life.	Psychosis as a potential result of attending to trauma.
Disclosure as the key to resolving alienation	Actions are inappropriate to realize intention.	Only when the protagonist stops hiding distress, better (trauma)care and social support initiate.	Trauma needs to be processed in order to rebuild life.	Trauma and psychosis as cumulative problems.

**TABLE 4:** Barriers and facilitators in the meaning-making process.

Story type	Meaning-making barriers	Meaning-making facilitators
Psychiatry as the wrong setting to find meaning	**Within mental healthcare** • Unsafety and seclusion. • Treatment limited to stabilization and medication. • Stigmatizing professionals: ○ Feeling treated as incapable of developing insight; ○ Feeling reduced to the illness.	**Outside mental healthcare** Peer-support: • Safety and respect; • Validation and exchange of experiential knowledge; • Self-inquiry through writing and creative self-expression.
The ongoing struggle to get trauma-therapy	**Outside mental healthcare** • Victim blaming and silencing of trauma. • Stigma and social exclusion related to psychosis. **Within mental healthcare** • Lack of recognition of traumatic stress. • Lack of trauma-focused psychoeducation. • Stigmatizing professionals: ○ Feeling treated as incapable of developing coping. ○ Feeling devaluated in meaning-making attempts. • Risk-avoidance: ○ Discouragement/refusal to treat trauma; ○ Strict inclusion criteria for trauma- therapy. • Lack of body-focused trauma therapy. • Lack of openness and innovation.	**Outside mental healthcare** • Supportive, non-judgmental individual, or (spiritual) community. • Self-education in mental health. • Self-inquiry through writing and creative self-expression **Complementary and alternative care:** • Spiritual meaning of distress. • Holistic, integrative approach. • Body-focused self-care techniques (e.g., breathwork). **Within mental healthcare** • Non-verbal, creative therapies. • Integrative, emotion focused psychotherapies (scheme, EFT). • Trauma-therapy (e.g., EMDR)
Exposure to trauma as a threat to a stable life	**Outside mental healthcare** • Fear of consequences of remembering trauma. • Difficulty retrieving and verbalizing memories. • Confusion about realness of traumatic experiences.	**Outside mental healthcare** • Peer support: • Hopeful role models; • New perspectives on coping-strategies.
Disclosure as the key to resolving alienation	**Outside mental healthcare** • Stigma expressed by next of kin. **Within mental healthcare** • Crisis admissions without follow-up care.	**Outside mental healthcare** • Love and responsibility for another being. **Within mental healthcare** • Trusted professionals • Trauma-therapy within multidisciplinary treatment.

**TABLE 5:** Narrative integration of trauma.

Story type	Characterization of trauma storyline	Causal coherence: continuity of self	Thematic coherence: reflection on self	Detailedness of trauma storyline	Recovery style: approach to distress.	Main form of disruption
Psychiatry as the wrong setting to find meaning	Part of self-story.	Medium: reconstructed continuity of self in light of past, excluding childhood.	High	Low	Combination of moving toward and turning away from distress.	Narrative dominance (resisted) and narrative dissociation
The ongoing struggle to get trauma-therapy	Central to self-story.	High: reconstructed continuity of self in light of past.	High	High	Moving toward distress.	Narrative dominance (resisted).
Exposure to trauma as a threat to a stable life	Gap in self-story.	Low: self is “stuck” in present, disconnected from past.	Low	Low	Turning away from distress.	Narrative dissociation and narrative dominance.
Disclosure as the key to resolving alienation	Unfinished storyline within self-story.	Low: in process of reconstructing continuity between ill and normal self.	Medium	Low	In transition from turning away to moving toward distress.	Disintegration of self.

### 3.1. Psychiatry as the wrong setting to find meaning (*N* = 7)

Narrators of these stories had all engaged in peer-support groups and were often working as experts-by-experience. Most of them appointed psychiatric treatment-related experiences as traumatic.

#### 3.1.1. Plot

These stories were mainly about the narrators’ quest to find meaning in their suffering. The plot was centered around the friction between this intention and the setting of mental healthcare, where the narrator initially expected to gain self-insight, but became disillusioned.

*“At first I was hopeful to get help within that mental health system. So I was very willing to cooperate, also in diagnostics. Of course I was walking around with a hundred thousand questions myself: What is going on with me? Why is it escalating like this? Why am I reacting to certain things? And as time went on and not so much was done with that, my candor also kind of stopped. Um, and, well, I also became suspicious (*…*) because you feel you are being observed, but nobody reports back to you*… *So that felt not really safe for me either.”*

“Mark” (47 years old).

Acute care settings were particularly experienced as unsafe and neglectful spaces, where previous trauma was triggered, or new trauma created. Narrators eventually found a setting that better supported their needs. In some cases, this entailed switching to another mental healthcare institution. In most of the stories, however, the “right setting” was found outside of mental healthcare, in a peer-support setting.

*“When I went to the recovery college, it was like getting a warm shower. They operate on the basis of equality. Well, you notice that right away when you enter. When you step inside, you already have such an idea of hey, what a nice atmosphere. It has been a second home for me. And I also learned a lot there, also personally. So that helps me enormously, you know. Because you feel equal, equally taken seriously. You are treated as a human being, just as eh, a full human being, so to speak.”*

“Ria” (60 years old).

#### 3.1.2. Meaning of trauma within the self-story

Narrators of this story type talked of trauma as both a cause and a consequence of psychosis. Although many narrators mentioned youth trauma, traumatic experiences within care were foregrounded in these stories. Overall, narrators articulated their suffering more strongly in terms of loss-experiences, than in terms of trauma. They described how illness- and treatment experiences had negatively affected their life course, leading to an accumulation of losses, mostly in terms of interrupted education, career and relationships. Often, these loss-experiences resulted in a feeling of being a failed or “depreciated” member of society. In their recovery process, trauma became part of their explanation for a disrupted life, thereby facilitating a more compassionate self-judgment. Some narrators described an ongoing process of trauma processing and intended to engage in trauma-therapy. However, for most of them the acknowledgement of their trauma history seemed most important.

*“To accept that formerly you had an incredibly nice job, a nice house and I don’t know what, and that you just get written off [by the occupational doctor red.]. That’s what it comes down to. And if you then also go through a divorce, which means you have to um, leave your house behind and your whole past in fact (*…*) then you have to work very hard to get through that. And, and, well, make a new story for yourself, of who you are and what happened to you and, and why and how that happened and where you stand now (*…*). And I do have an explanation in retrospect as to why I was so explosive at the time. Because the situation in which I regained consciousness [after an overdose, red.] was exactly the same as when I regained consciousness at the age of 5, after being in a very serious car accident. So that, that can be, traumatic. That, that’s my explanation (*…*) I came to the conclusion that it had everything to do with myself, with my life story.”*

“Jonathan” (71 years old).

#### 3.1.3. Barriers and facilitators in the meaning-making of trauma

Several aspects of the mental healthcare setting were experienced as barriers in the meaning-making process. Firstly, narrators reported experiencing how the predominant feeling of unsafety elicited by acute mental healthcare settings, led to further withdrawal, distrust and isolation, instead of dialog and meaning-making. (Coercive) admission and the seclusion it entailed, was often experienced as traumatic, and as causing a downward spiral of mental distress.

*“And um, then in 2001 I was isolated for the first time. And that really um, that really took me a long time to get over that. That really put the whole- I always call it ‘the revolving door of psychiatry’- in motion (*…*). It is like it fueled a kind of separation anxiety. So, I really didn’t dare to be alone anymore. Only when I was stoned or drinking, I could be with myself, alone.”*

“Maya” (52 years old).

Second, a short-term medical approach to their suffering was experienced as hindering. Although most narrators continued to make use of psychiatric services, they felt that their treatment had been too narrowly focused on symptom-reduction, by exclusively providing crisis admissions and medication. Third, narrators experienced stigma in mental healthcare. They felt reduced to their illness and approached as incapable of developing self-insight. For instance, because they were not asked about their life-history and professional insights were not shared with them.

In contrast, peer-support groups were experienced as the most important facilitator in the quest for meaning. Peer-support settings were experienced as safe spaces, were everyone was treated with equal respect. These basic conditions set the scene for enhancement of self-insight through mutual validation and exchange of experiential knowledge. Developing a meaningful self-story was an explicit aim of these groups. In this process, narrators experienced writing and creative self-expression as helpful means.

#### 3.1.4. Integration of trauma

Characteristic of these stories was a combination of high thematic coherence with a low degree of detail of traumatic events and experiences. Narrators clearly reflected a lot on their selves and lives and had experienced the benefits of integrating past-experiences into their self-story as part of their recovery process. Narrative dominance of the stigmatizing idea that people with psychosis are not able to make-meaning had hindered them, but was resisted through these counter-narratives. Although narrators had created causal coherence between who they were, how illness and care experiences impacted them, and how this affected their future intentions and (im)possibilities, in most stories there was limited elaboration on the traumatic childhood experiences they mentioned. They tended to seal over those experiences in the context of the interview: traumatic events and the feelings and thoughts they evoked remained vague.

### 3.2. The ongoing struggle to get trauma-therapy (*N* = 5)

Narrators of these stories had all experienced psychical assault, including sexual abuse.

#### 3.2.1. Plot

The quest for trauma-therapy was central to these stories. Protagonists emerged as active agents that put a lot of effort in gathering the right means to process trauma. They developed specific ideas about how trauma affected them and what kind of trauma-therapy they would need. However, their quest was hindered by mental healthcare professionals that refused or discouraged trauma-therapy or did not offer adequate support.

*“So, when it comes to medication and having confidence in my recovery, I definitely appreciated him [psychiatrist, red.]. But when it comes to explanatory models and what, what you can do*… *I also asked him repeatedly: What can you do for early childhood trauma? And then he said well, we don’t have any treatment for early childhood trauma. So, I can’t offer you anything. Then I went to the GP to refer me for EMDR for early childhood trauma (*…*). And yes, we’ve approached something like five or six practitioners. But they didn’t respond, or they said well, we think the risk is too high, because you are psychosis prone.”*

“Marjan” (51 years old).

*“They first helped me with creative therapy. Then I could be referred for EMDR and confrontation therapy. But I dissociate sometimes (.) and hyperventilate, and sometimes um, it gets so intense that I get a kind of epileptic seizure. So, I um, want to get through it, but I also asked for help -in case I would have such a seizure- to get out of it. But they wouldn’t, because they’re not going to hold your hand, that’s how they told me. So, it turned out I would be sent home in an epileptic seizure. But that’s not possible, you understand? I can’t handle that. So, I do need the EMDR and, and exposure therapy, but not that way.”*

“Roos” (48 years old).

Excluded from the desired trauma-therapy, protagonists often turned to alternative and complementary care. Although protagonists sought their own ways of treating trauma, they continued to struggle with trauma consequences, especially with physical stress reactions.

#### 3.2.2. Meaning of trauma within the self-story

Trauma was central to these self-stories. For narrators, there was a clear relationship between trauma and psychosis. They understood psychosis as both a consequence of trauma and a coping mechanism: an “escape” or “flight” from unbearable traumatic pain. Distinctive for these stories, and apparently related to the experience of physical assault, was that trauma experiences were talked about primarily as bodily experiences that kept inhabiting their bodies and elicit fear. Voices or visions were seen as having meaningful content and realistic elements, related to past trauma. Recovery from trauma for these narrators meant to be able to confront and process trauma without having to escape into another reality. Narrators were hopeful that once trauma was processed, psychosis would no longer occur.

#### 3.2.3. Barriers and facilitators in the meaning-making process

Negative responses of parents and other family members on traumatic events were important initial hinderers that narrators encountered in the aftermath of (childhood) trauma. They described interaction patterns of silencing, denial, neglect, or victim-blaming and condemnation, for example in cases were sexual abuse came out. Consequently, narrators came to feel guilty and ashamed about their experiences. Stigma became a barrier once their mental distress became visible for others. Narrators felt that their troubled behavior was not comprehended by others, leading to further despair. Traumatic experiences underlying their distress remained unexplored in mental healthcare, and traumatic stress was not recognized. Once they had been diagnosed with psychotic disorder narrators felt reduced to the “crazy” or “troublesome” person, whose attempts at meaning-making were devaluated and seen as a symptom of illness.

*“So, you’re there, with post-traumatic stress disorder and pain, neuropathic pain disorder. And you’re not being helped for that. Um, also eventually um, yeah, I got the report eventually. Yeah, nothing at all had been written about a violent crime [which he reported, red.]. So that information, nothing of it was written down there (.) And yeah, then you end up standing outside, because you’re not getting anywhere. Eventually you’re taken out of your house by the police about ten times, or picked up from the street. Um, [silence] because I did start drinking a lot. And [silence] so then you’re actually taken out of your house. And you start to wish: if only I were dead.”*

“David” (47 years old).

Related to (self-)stigma was the lack of psychoeducation on trauma-related coping mechanisms, particularly on dissociation: Narrators thought this information could have helped them in an earlier stage to understand their experiences not as crazy, but as normal reactions to abnormal circumstances.

*“When I was abused as a child, I found myself in another place. In my mind it wasn’t me that it had happened to. Much later, I learned that this is called dissociation. But as a child, I felt very strange: suddenly you open your eyes, where am I? So, I didn’t understand what happened to me, but I also didn’t know who to talk to, because I just thought I was crazy. So, I think a lot of mistakes were made there, and that if I would have had better guidance, I could have been spared for what came next.”*

“Roos” (48 years old).

An important turning point in these stories was the encounter with a supportive, non-judgmental partner, professional or community that made narrators feel accepted for who they were, beyond their problems. In particular, spiritual or religious communities played an important role by acknowledging the meaningfulness of their distress. Feeling more motivated and hopeful, narrators started to engage in a process of self-inquiry and education on mental distress, finding out more about trauma-related mechanisms, coping and treatment options. Some of them started to write and talk about trauma, others could not speak about it initially, but found it liberating to express themselves through dance, painting or other creative means.

Once narrators were able to verbalize their trauma history, trauma, and in most cases PTSD was acknowledged in mental healthcare. However, new barriers arose at this point. Most importantly, narrators felt hindered by risk-avoidance, both as an attitude of individual practitioners and an institutionalized practice in the form of strict inclusion and exclusion criteria for trauma-treatment programs, disadvantaging people with complex psychopathology. They were particularly confronted with the assumption that people with psychosis are not able to (learn to) cope with trauma.

*“If disasters happen, well, there’s a whole trauma team ready to help the victims. Well, for me, there wasn’t. And during that period when I was psychotic, um, I think there was also the belief that people who are psychotic couldn’t do trauma processing, because they were afraid that they would become psychotic again. Well, that’s bizarre anyway. So, you go through something very shocking [silence] and everybody actually says: don’t talk about it.”*

“Jeanet,” (52 years old).

As narrators educated themselves on trauma and psychosis, they felt hindered by the slow pace in which new scientific insights were integrated in guidelines and translated into practice. Within the mental healthcare that was accessible to them, narrators experienced integrative psychotherapies (such as scheme therapy) as beneficial, especially in terms of validating and regulating emotions associated with trauma. Some narrators finally had the chance to engage in EMDR for a single traumatic experience, that helped them to process it. However, a problem for most narrators was that they had multiple traumatic experiences, and that childhood trauma in particular, was found impossible to verbalize or translate into concrete images. Associated, the lack of innovation and body-focused approaches in mental healthcare was experienced as a barrier. In alternative and complementary care, narrators felt helped because of the holistic approach that facilitated body-mind integration. Learning different self-care techniques, such as breathwork, meditation or reiki made them feel more agentic in calming down their bodies. This made narrators more confident in their capability to further confront and process trauma.

*“My whole body gets in a kind of panic or something, and starts to shake all at once. So that’s one big startle reaction. Especially when I’m touched, I can’t stand that anymore. I um, [silence] I can handle it a little bit, but it’s not gone away yet (.) Eh, but that’s still um. I just think it’s unfortunate that I just can’t get real professional help within the mental health system, can’t get on with that (*…*)”*

*I did a lot of things like shiatsu massage. Or um, I’ve done other. I did the whole training on shamanism. That also helped me a lot (*…*). They’re especially good at early childhood trauma. Because there’s almost always dissociation. And that’s where I also suffer from: dissociative thoughts. And, well, what a shaman is trained in, is to figure out where these experiences went. And how can you get that back again so to speak (.). So, I thought, if I make the technique my own, then I also don’t depend on others. Then I can apply it to myself.”*

“Marjan” (51 years old).

#### 3.2.4. Integration of trauma

Clearly, trauma was central to these stories. Unlike the other story types, the self-story began with the first traumatic experience, not with the onset of illness. Narrators gave elaborated accounts on trauma, the way they coped with it and its impact on their identity. Distinctive was that narrators described many traumatic events in detail, including the ambiguous and confusing emotions and thoughts that they had experienced during these events. Both causal and thematic coherence were high in these stories. Given the efforts of narrators to inquire and process trauma, this was not a surprising finding. However, narrators emphasized that making meaning of trauma had been a difficult process that had often taken them several decades. Their highly integrated trauma stories can be read as a form of narrative resistance to the idea that people with psychosis are unable to make meaning. However, narrative dominance of the idea that people with psychosis are unable to cope was still hindering them in their quest for trauma-therapy and further body-mind integration.

### 3.3. Exposure to trauma as a threat to a stable life (*N* = 5)

Narrators of these stories were all single persons that had recently started to participate in (online) peer-groups or rehabilitation trajectories. This subgroup included the three stories of people who described traumatic experience without labeling it as such.

#### 3.3.1. Plot

Friction between different intentions was central to this story type. Narrators typically had been focusing successfully on keeping their psychotic symptoms under control and avoiding relapse and psychiatric admission. Medication, living a tranquil, withdrawn life, and focusing on the here and now had been their most important means to achieve a state of stability. However, at the downside, this lifestyle made them feel isolated from other people and society. Influenced by exposure to new ideas about recovery from peers, narrators began to hope for a more meaningful life. At the same time, they felt anxious about taking new steps that could trigger trauma and compromise their stability.

*“Um, well, I really want to study actually. But I’m not up to it yet (…). That has to do with um, crowds, stimuli and people and um, fears. I had a bad time in high school. I’ve been bullied for several years. So um, I’m afraid that I’m going to end up in a situation um, yes, that I’ve also experienced in the past. I feel, already feel very vulnerable thinking about that.”*

“Tim” (40 years old).

*“I would like to have a job, being able to raise children. Even if it’s just a job for ten hours a week. Even if it’s volunteer work. Doesn’t matter to me. As long as I’m just stable and can get to work. That’s actually the most important, because then I can feel part of society. Then I can also make an active contribution (*…*). They say I should just do things. But you’re just scared every day that things can go wrong again. That you’ll be locked up again. That fear is so hard to live with.”*

“Sanne” (22 years old).

#### 3.3.2. Meaning of trauma within the self-story

Narrators associated preoccupation with past trauma with periods of mood disorder and psychotic deregulation. Hence, they believed it was best for their well-being to not look into trauma. They had actively turned away from their past and apparently sought shelter in the present.

#### 3.3.3. Barriers and facilitators in the meaning-making process

In these stories, hesitance and difficulty in meaning-making of trauma seemed intertwined. Narrators feared the consequences of attending to trauma. At the same time, they showed difficulty in retrieving and verbalizing memories. Some narrators explicitly mentioned this difficulty. From the evaluation forms of the interviewers, we learned that these interviews were often experienced as demanding and requiring a more active and structured interview style. An apparently related but distinctive hindering factor was narrators’ doubt and confusion about the “realness” of their traumatic experiences, describing their memories as being “blurred,” “diffuse” “delusional” or even “false.”

*“Before, I was living very much in the past. Um, I had to think very often about my um, early childhood which went rather oddly. But they were partly real memories and partly false memories. And um, yes, I’ve lost that now. I live more in the here and now.”*

“Bas” (46 years old).

Helpers were scarce in these stories, however one important and consistent helper across these stories was peer-support. Firstly, because peers offered narrators hopeful perspectives on recovery in psychosis, motivating them to explore new future possibilities. Secondly, examples of how others were dealing with trauma made them begin to reconsider their own strategies. Thus, peer-support seemed particularly helpful in offering role-models.

*“I’m thinking about*…*maybe taking some kind of course to write a book or something. That would be good thing. I have two classmates who have written a book and they motivate me to do it too. But then I don’t really know how. I really wonder how I can deal with my pain, cause I’m kind of overflowed. When I go out with my friends, I’m always cheerful, and I think more about my future nowadays (*…*). I don’t look at that pain so much. I know that the pain is there, that it does hurt. But then I wonder: what should I do with that? I find that difficult. Some people may be able to deal with that in their own way. But then I am not so much looking at the pain.”*

“Dolores” (38 years old).

As narrators of this story type were not directed toward meaning-making of trauma, barriers and facilitators were harder to identify and interpret in these stories. The data-validation session with experts-by experience played an important role in filling in the gaps of these stories and our understanding of them. They brought forward three important points, based on their own experiences. Firstly, that reluctance to speak about trauma might arise from not being aware of the benefits. Trauma might be perceived as unchangeable life fact that can’t be made “better.” Secondly, they suspected that the fear of trauma memories might consist mainly of losing control over disturbing emotions associated with these memories, such as grief or anger. Thirdly, they suspected that feelings of guilt and loyalty might be involved in the doubt about the realness of traumatic experience. For example, in case of (youth) trauma in which loved-ones are involved, acknowledging the experience as traumatic turns loved-ones into perpetrators. A lack of witnesses to confirm or validate the traumatic experience was brought forward as another factor that can enhance doubt about the realness of it.

#### 3.3.4. Integration of trauma

These self-stories were mostly centered on illness experiences. Traumatic experiences were the gaps in these stories. Narrators mentioned traumatic experiences indirectly and between the lines, referring for example to “bad” situations or experiences without providing further details. Only when they were invited by the interviewer to elaborate, traumatic experiences became somewhat more explicated. These elaborations were minimal and remained low in causal and thematic coherence. These stories were characterized by narrative dissociation of trauma, a form of disintegration in which trauma-memories seem blocked and (unconscious) psychological defense mechanisms were most clearly at work. At the same time narrators’ stories appeared to be overruled by narrative dominance of a stigmatized illness-identity.

### 3.4. Disclosure as the key to resolving alienation (*N* = 4)

Narrators of these stories were females within similar care-contexts: They all started with trauma-therapy (preparation), as part of a multi-disciplinary treatment.

#### 3.4.1. Plot

Central to this story type was past friction between the protagonists’ intentions and actions. When mental distress started to interfere with their pursuit of a “normal” life, protagonists believed it was best to hide their distress. They took actions that consisted of fleeing physically (e.g., running away from their family or institution) or mentally (e.g., substance abuse to numb the pain, blocking memories, pretending to be okay).

*“I had seen something on TV, a program about schizophrenia. And I did realize that I had that too. And I was afraid they were going to lock me up. And, so I though, either I have to act totally normal or I have to end my life. I had those two choices. And I decided to go back to normal. And that actually worked out at that time. I called off my psychiatrist, threw away my pills and I just went back to living a normal life. Even finished my studies. And then things actually went really well for quite a while (*…*). I didn’t even remember having had it [psychosis red.] and hence never told my husband about it. Until I got it again.”*

“Bea” (44 years old).

However, on the long term these actions led to a downward spiral of problems (such as divorce, loss of custody, forced admissions) and a sense of alienation of their selves and loved ones. Only when the protagonists stopped hiding their distress and found the courage to disclose traumatic experiences, better care and social support initiated.

*“I didn’t dare to talk openly about what I had gone through (*…*). I felt ashamed of the things I had been through. I didn’t want to be pitied and that kind of stuff. And [silence] um, yeah, if you don’t share who you are then you don’t have real relationships either. The appearance, the mask you put on, that jams with the others. But a real conversation where you share your heart and where you show who you are, and the other [silence] can respond to that, I just didn’t have that for years. And then you alienate yourself. And I feel now that really um, clarity and light has come to me um, to the things that I was all tucking away.”*

“Sarah” (60 years old).

#### 3.4.2. Meaning of trauma within the self-story

Narrators described how the role of trauma in their lives had recently shifted. For a long time, they had seen it as something to be hidden for themselves as much as for others. When this strategy turned out to obstruct, rather than help to live a normal life, their ideas changed. They had come to see trauma as an experience that needs to be disclosed and processed, in order to be able to rebuild their lives. Their wish for a better future became the motivation to address the past.

*“I’ve also experienced trauma in my life*…*In the sexual area, when I was very young. Now I can talk about it. And talking also means processing. But in the beginning when I just got that first psychosis, I didn’t dare to talk about it. With my psychologist, we are looking now at some form of trauma treatment. We explore what suits me best and how I can get on top of it (*…*). I especially want to learn how to cope with the thoughts that came after the trauma. And I also um, want to stay stable to be able to take care of my daughter again.”*

“Alissa” (25 years old).

Narrators did not articulate particular ideas about the relation between psychosis and trauma but spoke of psychosis and trauma as accumulative problems.

#### 3.4.3. Barriers and facilitators in the meaning-making process

In these stories, next of kin played a particular negative role in the meaning-making process, by expressing stigmatizing ideas on mental distress. Protagonists felt rejected by their family or partners, making them feel ashamed and afraid to disclose their inner world.

*“For a very long time I thought I’m going to be the old one again. And my family also hoped that very much. They were waiting for the day that I became my old self again. But I didn’t, I already knew that. Um, and I really became a new person. And for a very long time I didn’t dare to come out (*…*). And for a very long time I was ignored and not allowed to be there as a person. During the admission, nobody came to see me either, because I wasn’t the person they wanted to see.”*

“Bea” (44 years old).

*“I think psychosis it is a very hard, lonely disease that you are condemned for (…). Um, [silence] yes, you, you can’t imagine that there’s really no one at all who thinks well, let’s go and see her. And that for two and a half years. It feels like you’re already dead, only you’re still breathing.”*

“Marlies” (53 years old).

Acceptance of professional help after subsequent losses was an important turning point in these stories. Notably, narrators’ motivation to accept and commit to care was the love and responsibility for another being—a child, God, or even a pet—that gave them the strength to work on recovery and start rebuilding their lives. (Coercive) crisis admissions was described as a hinderer, unless it was embedded in trustful therapeutic relationships and follow-up care. Trauma-sensitive care within a multidisciplinary team was described as a significant helper in the meaning-making of trauma. Unique for this story type was that narrators were actually offered regular trauma-therapies, such as EMDR and imaginary exposure. Narrators felt helped by the involved and cooperative relationship with -and between members of the team, and the integrated manner in which various problems were addressed. Marlies, for example, described how she told the psychologist of her Flexible Assertive Community Treatment (FACT) team that the side-effects of her medication made her too passive to feel motivated for EMDR. Hence her psychologist agreed with her psychiatrist to lower the dose. At the same time, the psychologists involved Marlies’ parents to restore her lost supportive network. Simultaneously, a social worker helped Marlies with daily chores and prevention of eviction by the house cooperative. Thus, the whole team together created the right conditions for effective trauma-therapy.

#### 3.4.4. Integration of trauma

The integration of trauma in these stories is best characterized by their transition. Narrators had moved away from trauma for a long time but were starting to turn toward it. Most of their trauma storylines were not detailed or elaborated yet. However, narrators created thematic coherence by stressing the significant impact of trauma on their lives, and the necessity and intention to process it. Hence, the storylines had begun to be part of the self-stories but were unfinished. One exception was the story of Sara, with a highly integrated and elaborated trauma storyline. In contrast to the other narrators that were beginning trauma-treatment, Sara had already finished trauma-therapy, and experienced the effect of it.

*”I am very happy with the help that I receive now, and the imaginary exposure therapy I got. And that um, [silence] yes, that I just worked through the pain. Because before, I was always tired. A lot of energy went into pushing away that pain. And now I have that energy left and I feel much better than when I was 20, both physically and psychologically. And that is really a wonderful experience.”*

“Sarah” (60 years old).

A salient characteristic of these stories was the relatively low causal coherence: narrators struggled to integrate ideas of their present and past self. After long periods of radically hiding mental distress, the creation of a story that was able to restore a sense of continuity took them considerable effort. Disintegration of the self characterized this story type. Narrators articulated discontinuity specifically in terms of the self before and after psychosis. Experienced stigma played an important role in this form of disintegration, as the ill-self could not be accepted as part of the self without losing significant others. Unlike in other story types, mental healthcare professionals played an important and destigmatizing role in the meaning-making of both illness and traumatic experiences.

## 4. Discussion

The aim of this study was to gain in-depth insight into how people with psychosis make-meaning of trauma. Furthermore, we wanted to understand how adaptive meaning-making, as part of the personal recovery process, can be better supported in mental healthcare.

Meaning-making was studied by performing storyline analysis on 21 narratives of people that experienced psychosis and trauma. We identified four story types within our sample, each entailing a different meaning of trauma in the context of the self-story. Narrators of “Psychiatry as the wrong setting to find meaning” spoke of trauma as both a cause and consequence of psychosis. They articulated their suffering mostly in terms of illness- and treatment related trauma and losses. The acknowledgement, rather than the processing of trauma seemed most important for them. Distinctively, narrators of “The ongoing struggle to get trauma-therapy” had come to see psychosis as a way of coping with the unbearable pain of traumatic experiences. Consequently, confronting trauma became vital for their recovery. By contrast, narrators of “Exposure to trauma as a threat to a stable life” associated psychotic crisis with preoccupation about past trauma. They appeared to get stuck in the present, as avoidance prevented the integration of trauma into the self-story, but simultaneously hindered them in creating future possibilities. Lastly, narrators of “Disclosure as the key to resolving alienation” had concluded that their strategy to radically hide mental distress and its traumatic origins was not viable. They spoke of addressing trauma as a necessary step in the rebuilding of their lives. These outcomes are consistent with previous qualitative studies, indicating that people with psychosis often link experiences of illness to trauma (29, 30) and expand insight into meaning-making differences.

We also found that most narrators either demonstrated or reported (past) difficulty integrating trauma. Both illness and trauma are disruptive experiences that can translate into disintegrated self-stories (41). In this study, we identified stigma as a major additional contributor to narrative disintegration. From the narratives, we learned that stigma was experienced in different contexts, but especially within mental healthcare. In particular, narrators encountered two stigmatizing ideas that hindered them in their meaning-making efforts: The idea that people with psychosis are not able to develop adequate self-insight and meaning, and the idea that they are not able (to learn) to cope with distress. Narrative dominance of these ideas appeared to be related to other experienced barriers in mental healthcare, such as lack of access to trauma-therapy and little attention for the trauma history. The disrupting influence of stigma within mental healthcare was most clearly articulated by narrators of “Psychiatry as the wrong setting to find meaning” and “The ongoing struggle to get trauma-therapy.” Narrators of the latter story type felt that their attempts to make meaning were actively undermined or rejected in mental healthcare, whereas narrators of “Psychiatry as the wrong setting to find meaning” felt that mental healthcare lacked the characteristics and resources to develop meaningful narratives at all. Similar service-user experiences have been conceptualized as ways in which mental health services can undermine autobiographical power of people with psychosis (75). Narrators of the above story types managed to regain autobiographical power, outside and despite mental healthcare. Others however, found support within mental healthcare: In “Disclosure as the key to resolving alienation,” next of kin were the stigmatizing actors, whereas mental health professionals came forward as helpers in the creation of a more viable an empowering meaning of both illness and trauma. Narrators of “Exposure to trauma as a threat to a stable life,” with the least integrated trauma storylines, appeared to suffer most from internalized stigma and social isolation. They had only just begun to discover new perspectives and future possibilities in interaction with peers. These differences stress the significance of supportive networks and discursive resources in the transition from moving away, to moving toward distressful experiences [see (57)].

### 4.1. Implications for practice and education

To offer meaning-specific entries for personalizing trauma-informed care, we analyzed barriers and facilitators of each story type in detail. Here we will discuss three overarching patterns that offer guidance to improve trauma-informed care for people with psychosis: the importance of better relational preconditions, long-term care and access to psychotherapeutically interventions.

First, we learned that a process of adaptive meaning-making often started with the experience of supportive, non-judgmental relationships with individuals or communities. In accordance with findings from Campodonico et al. (33), feeling respected and safe was often spoken of as a novel experience that motivated people to engage in a process of self-inquiry and growth, including the disclosure and exploration of traumatic experience. We also learned that supportive relationships were often found outside mental healthcare: Partners, peers, spiritual communities and alternative care providers were appointed as important helpers. Clinicians may more actively encourage engagement with such helpers, but can also learn from the healing qualities of these relationships, such as equality. Clinicians can express stigma subtly and unintendedly to people with psychosis (76). Hence, it seems crucial to raise awareness of stigmatizing practices and their consequences as part of their professional education and training in order to create better relational preconditions.

Second and interrelated, our outcomes highlight the importance of long-term care. Building supportive relationships requires an organizational context that allows professionals to be attentive and stay present (77). Instead, the acute care settings where people with psychosis often find themselves are literally characterized by seclusion. In correspondence with previous studies (15, 16, 31), many narrators in our study described psychiatric admissions as traumatic. Such traumatic treatment experiences can damage patients’ trust and hinder engagement with mental health services (16). The way acute care was followed up appeared to be of utterly importance to mitigate negative care experience. People that felt most hindered by mental healthcare often described crisis admissions that were merely followed up by medication controls. By contrast, people that felt supported by mental healthcare reported to receive continuous, integrated care by multidisciplinary teams with familiar professionals. Although the latter is actually in line with quality standards, people with psychosis—especially those from marginalized groups—are at risk to receive only minimal care (78).

Third, the outcomes highlight the importance of better access to psychotherapeutically interventions. Narrators experienced a lack of access to interventions that foster meaning-making in general—such as psycho-educations and psychotherapy–as well as access to specific trauma-focused therapies. Psychosis, by definitions consist of a “breakdown of shared meaning” (79), and may cause clinicians to be reluctant in the provision of psychotherapy. They can, however, play an important role in supporting the process of finding more viable and socially shared meaning. Several therapies, such as Open Dialog (80) and Metacognitive Reflection and Insight Therapy (81) have been developed for this purpose. Additionally, our outcomes stress the importance of creative, body-focused, and emotion-focused approaches to integrate trauma in non-verbal ways.

Education on psychological mechanism- especially that of dissociation-came forward as another helper in creating a more viable and destigmatizing meaning of trauma reactions. The illness-focused psychoeducation that is common in mental healthcare can be helpful in the acceptance of mental distress and professional help. However, in the case of trauma in psychosis, this approach might be problematic, both because of underdiagnosed PTSD and the stigma-related negative effect of “illness insight” in psychosis (82, 83). Additionally, being told that your experiences are unreal can be a very disorienting experience (21). People that are confused about the realness of their experiences might benefit more from psychoeducation on the -often decontextualized- phenomenological similarities between the content of hallucinations and traumatic experiences (84–86). This information may help them to connect the dots between traumatic life events and alienating psychotic experiences.

Post-traumatic stress in people with psychosis has been found to be systematically underdiagnosed and undertreated in mental healthcare. In our study, few participants had experience with trauma-focused therapies, such as EMDR and exposure therapy. As we learned, people that suffer from trauma consequences will not always seek help for it. Clinicians can play an important role in encouraging people to engage in therapies that reduce the avoidant behavior that is part of post-traumatic stress reactions. Unfortunately, research indicates that in the case of people with psychosis, avoidance might actually be sustained by clinicians, as a consequence of their own negative expectations or anxiety (87–89). This pattern is aptly described by Boevink (90) as a dialogical process of not wanting to, and not being allowed to look at the dark sides of the self, informed by the idea that talking about distress will aggravate psychosis. In our study, we found that even people willing to confront trauma, had difficulty finding clinicians and institutions willing to provide trauma-therapy. In this way, risk-averse mental healthcare systems can undermine actions of positive risk-taking that can contribute to recovery (91). Specialized training has been found to effectively decrease clinicians’ negative expectations about trauma-focused therapy in people with psychosis (88). Furthermore, guidelines and practices are changing in accordance with the accumulating evidence that trauma treatment is effective and safe in people with psychosis (92–96). Our results stress the urgency to accelerate implementation of trauma-therapy for people with psychosis.

### 4.2. Contributions and limitations

Although meaning-making of trauma is an important element of recovery in psychosis (27), studies that offer in-depth insight into this meaning-making process are scarce. In this study, we made use of a qualitative, narrative methodology that allowed us to obtain both a differentiated and contextualized picture of meaning-making efforts of people with psychosis. In the (quantitative) narrative identity literature, meaning-making of people with psychosis has been characterized in terms of deficits. Yet contextualization of these deficits is often lacking. For instance, based on a review, Cowan et al. (53) proposed a developmental model of narrative identity in psychosis, in which confusing psychotic experiences cause people to give up their attempts to make sense of their lives. However, this process is exclusively interpreted in terms of individual, psychological mechanisms. The outcomes of our study highlight the importance of social relationships in determining whether meaning-making attempts fail or flourish and demonstrate the devastating effect of stigma on this process.

Another strength of this study is the use of low-structured interviews, that permitted us to inquire the spontaneous integration of trauma into the self-stories of people with psychosis. In this way, we were able to identify a broad variety of meaning-making repertoires: Previous qualitative research has mostly focused on how people with psychosis make meaning of illness experiences and resulted in distinction between people that seal over illness experiences and people that integrate illness experiences. In relation to trauma, we found more variation and combinations, such as highly integrated and reflexive self-stories with a lack of integration of childhood trauma. Insight in these differences can help mental health professionals to attune to particular challenges in the meaning-making of trauma.

Furthermore, insight in the different perspectives of people with psychosis can inform re-conceptualizations of trauma in psychosis. Research on psychosis has become increasingly guided by theoretical application of trauma models (9). It is important to validate and improve such models in dialog with the “inside perspectives” that qualitative studies illuminate. The perspectives brought forward by our participants are in line with the idea that the interplay between trauma and psychosis is more complex than the most studied link between childhood trauma as a predictor for later psychosis (8). Within our small sample, all three pathways as proposed by Morrison et al. (6) were represented in the different story types. Our findings stress the need for taking into account that illness and treatment experiences are potentially (re)-traumatizing (69). As Stevens et al. (8) argue, a lack of recognition of “psychosis -induced PTSD” may be a powerful maintenance factor for chronicity and relapse of symptoms.

In order for trauma models to be valuable for patients, they also need also to be profoundly grounded in an understanding of what people with psychosis struggle with in their lives. In line with findings from a qualitative study of Vallath et al. (32), our outcomes indicate that some people with psychosis suffer more from the accumulating losses that they experience, than from the consequences of “classical” traumatic events. These losses include relationships with significant others, but also loss of social status and dignity caused by the stigma on psychosis. Such experiences have been elaborated in the social defeat theory of psychosis, which conceptualizes the repeated experience of social failure, or of being put down by powerful others, as both a risk factor and a consequence of psychosis (78, 97). Emerging evidence indicates that experiences of social defeat mediate the link between trauma and psychosis (98). Our study further supports the plea for better inquiry and integration of these “social pathways” in theories of trauma and psychosis [see (9)].

Limitations of our study include issues of representation. The majority of participants were female, middle aged, highly educated and participating in society. Under-represented groups included people with a migration background, that have been found to be at higher risk for psychosis and associated experiences of social defeat (99). Unintendedly, these representations issues may lead to the reproduction of inequalities in narrative power (100). Furthermore, the Psychiatry Storybank is a project that aims to improve psychiatric services, with the help of experiential knowledge. Participants enroll on their own initiative. Possibly, our project is more appealing for service-users with negative care experiences, as satisfied service-users might feel less urgency to participate.

Another limitation concerns trauma-assessment. In this study, we identified traumatic experience on the basis of the content of stories people told about themselves. Previous research indicates that traumatic experience does not necessarily become part of the self-story of people with psychosis (52). Storytellers can have difficulty verbalizing trauma, or feel the need to minimize or silence traumatic experiences to produce “acceptable stories” (101). Thus, it is likely that we excluded interviews of people that have experienced trauma but did not speak about it. Systematic assessment of participants’ trauma exposure could have helped to identify this subgroup. Additionally, a mixed-methods design including PTSD assessment, could be a valuable way of further elaborating meaning-making patterns in relation to post-traumatic stress severity, or different subgroups of trauma in psychosis (8).

## 5. Conclusion

This study demonstrates various ways in which people with psychosis make meaning of traumatic experience. Stigma in mental healthcare was identified as a major barrier in the meaning-making process, especially the ideas that people with psychosis are not able to develop self-insight and coping. The outcomes highlight the social context of the meaning-making challenges that people with psychosis face. Over the last decades, service-user oriented research has challenged pessimistic views on the possibilities of people with psychosis to recover and has elucidated the self-affirming effect of such ideas. Our results suggest that the same self-fulfilling mechanisms could be at play in the meaning-making of trauma and provides directions to better support this process.

## Data availability statement

The datasets presented in this article are not readily available because of participant confidentiality and privacy. Requests to access the datasets should be directed to NS, n.vansambeek@umcutrecht.nl.

## Ethics statement

The studies involving humans were approved by The Medical Ethical Review Committee of the University Medical Center of Utrecht. The studies were conducted in accordance with the local legislation and institutional requirements. Written informed consent for participation in this study was provided by the participants.

## Author contributions

NS: Conceptualization, Formal analysis, Investigation, Methodology, Validation, Writing – original draft. GF: Conceptualization, Funding acquisition, Supervision, Validation, Writing – review and editing, Methodology. SG: Funding acquisition, Supervision, Validation, Writing – review and editing, Conceptualization. FS: Conceptualization, Funding acquisition, Supervision, Validation, Writing – review and editing.

## Footnotes

1 Patient advocates have suggested “Psychosis susceptibility syndrome” as more recovery-oriented alternative.

2 Here we follow the American Psychological Association that puts more emphasis on the experience rather than de severity of the event.

## References

[1] George B Klijn A. Psychosis susceptibility syndrome: an alternative name for schizophrenia. *Lancet Psychiatry.* (2014) 1:110–1. 10.1016/S2215-0366(14)70249-4 26360566

[2] Bonoldi I Simeone E Rocchetti M Codjoe L Rossi G Gambi F Prevalence of self-reported childhood abuse in psychosis: a meta-analysis of retrospective studies. *Psychiatry Res.* (2013) 210:8–15. 10.1016/j.psychres.2013.05.003 23790604

[3] de Vries B van Busschbach JT van der Stouwe ECD Aleman A van Dijk JJM Lysaker PH Prevalence rate and risk factors of victimization in adult patients with a psychotic disorder: a systematic review and meta-analysis. *Schizoph Bull.* (2019) 45:114–26. 10.1093/schbul/sby020 29547958 PMC6293237

[4] Mauritz MW Goossens PJJ Draijer N van Achterberg T. Prevalence of interpersonal trauma exposure and trauma-related disorders in severe mental illness. *Eur J Psychotraumatol.* (2013) 4:19985. 10.3402/ejpt.v4i0.19985 23577228 PMC3621904

[5] Seow LSE Ong C Mahesh MV Sagayadevan V Shafie S Chong SA A systematic review on comorbid post-traumatic stress disorder in schizophrenia. *Schizoph Res.* (2016) 176:441–51. 10.1016/j.schres.2016.05.004 27230289

[6] Morrison AP Frame L Larkin W. Relationships between trauma and psychosis: a review and integration. *Br J Clin Psychol.* (2003) 42:331–53. 10.1348/014466503322528892 14633411

[7] Hardy A. Pathways from trauma to psychotic experiences: a theoretically informed model of posttraumatic stress in psychosis. *Front Psychol.* (2017) 8:697. 10.3389/fpsyg.2017.00697 28588514 PMC5440889

[8] Stevens LH Spencer HM Turkington D. Identifying four subgroups of trauma in psychosis: vulnerability, psychopathology, and treatment. *Front Psychiatry.* (2017) 8:21. 10.3389/fpsyt.2017.00021 28243211 PMC5303718

[9] Heriot-Maitland C Wykes T Peters E. Trauma and social pathways to psychosis, and where the two paths meet. *Front Psychiatry.* (2021) 12:804971. 10.3389/fpsyt.2021.804971 35082703 PMC8785245

[10] Stanton KJ Denietolis B Goodwin BJ Dvir Y. Childhood trauma and psychosis: an updated review. *Child Adolesc Psychiatr Clin North Am.* (2020) 29:115–29. 10.1016/j.chc.2019.08.004 31708041

[11] McGrath JJ Saha S Lim CCW Aguilar-Gaxiola S Alonso J Andrade LH Trauma and psychotic experiences: transnational data from the World Mental Health Survey. *Br J Psychiatry.* (2017) 211:373–80. 10.1192/bjp.bp.117.205955 29097400 PMC5709675

[12] Mayo D Corey S Kelly LH Yohannes S Youngquist AL Stuart BK The role of trauma and stressful life events among individuals at clinical high risk for psychosis: a review. *Front Psychiatry.* (2017) 8:55. 10.3389/fpsyt.2017.00055 28473776 PMC5397482

[13] Varese F Smeets F Drukker M Lieverse R Lataster T Viechtbauer W Childhood adversities increase the risk of psychosis: a meta-analysis of patient-control, prospective- and cross-sectional cohort studies. *Schizoph Bull.* (2012) 38:661–71. 10.1093/schbul/sbs050 22461484 PMC3406538

[14] Shevlin M Armour C Murphy J Houston JE Adamson G. Evidence for a psychotic posttraumatic stress disorder subtype based on the National Comorbidity Survey. *Soc Psychiatry Psychiatr Epidemiol*. (2011) 46:1069–78. 20812006 10.1007/s00127-010-0281-4

[15] Berry K Ford S Jellicoe-Jones L Haddock G. PTSD symptoms associated with the experiences of psychosis and hospitalisation: a review of the literature. *Clin Psychol Rev.* (2013) 33:526–38. 10.1016/j.cpr.2013.01.011 23500156

[16] Lu W Mueser KT Rosenberg SD Yanos PT Mahmoud N. Posttraumatic reactions to psychosis: a qualitative analysis. *Front Psychiatry.* (2017) 8:129. 10.3389/fpsyt.2017.00129 28769826 PMC5515869

[17] Lommen MJ Restifo K. Trauma and posttraumatic stress disorder (PTSD) in patients with schizophrenia or schizoaffective disorder. *Commun Mental Health J.* (2009) 45:485–96. 10.1007/s10597-009-9248-x 19777347 PMC2791484

[18] Read J Harper D Tucker I Kennedy A. Do adult mental health services identify child abuse and neglect? A systematic review. *Int J Mental Health Nurs.* (2018) 27:7–19. 10.1111/inm.12369 28815844

[19] de Bont PAJM van den Berg DPG van der Vleugel BM de Roos C de Jongh A van der Gaag M Predictive validity of the Trauma Screening Questionnaire in detecting post-traumatic stress disorder in patients with psychotic disorders. *Br J Psychiatry.* (2015) 206:408–16. 10.1192/bjp.bp.114.148486 25792693

[20] Grubaugh AL Zinzow HM Paul L Egede LE Frueh BC. Trauma exposure and posttraumatic stress disorder in adults with severe mental illness: a critical review. *Clin Psychol Rev.* (2011) 31:883–99. 10.1016/j.cpr.2011.04.003 21596012 PMC3148295

[21] Britz B. Listening and hearing: a voice hearer’s invitation into relationship. *Front Psychol.* (2017) 8:387. 10.3389/fpsyg.2017.00387 28352245 PMC5348518

[22] Sweeney A Filson B Kennedy A Collinson L Gillard S. A paradigm shift: relationships in trauma-informed mental health services. *BJ Psych Adv.* (2018) 24:319–33. 10.1192/bja.2018.29 30174829 PMC6088388

[23] Ng F Ibrahim N Franklin D Jordan G Lewandowski F Fang F Post-traumatic growth in psychosis: a systematic review and narrative synthesis. *BMC Psychiatry.* (2021) 21:607. 10.1186/s12888-021-03614-3 34865627 PMC8647418

[24] Anthony WA. Recovery from mental illness: the guiding vision of the mental health service system in the 1990s. *Psychosoc Rehabil J.* (1993) 16:11–23. 10.1037/h0095655

[25] Andresen R Oades L Caputi P. The experience of recovery from schizophrenia: towards an empirically validated stage model. *Austral N Zeal J Psychiatry.* (2003) 37:586–94. 10.1046/j.1440-1614.2003.01234.x 14511087

[26] Leamy M Bird V Le Boutillier C Williams J Slade M. Conceptual framework for personal recovery in mental health: systematic review and narrative synthesis. *Br J Psychiatry.* (2011) 199:445–52. 10.1192/bjp.bp.110.083733 22130746

[27] Wood L Alsawy S. Recovery in psychosis from a service user perspective: a systematic review and thematic synthesis of current qualitative evidence. *Commun Mental Health J.* (2018) 54:793–804. 10.1007/s10597-017-0185-9 29188393

[28] van Weeghel J van Zelst C Boertien D Hasson-Ohayon I. Conceptualizations, assessments, and implications of personal recovery in mental illness: a scoping review of systematic reviews and meta-analyses. *Psychiatr Rehabil J.* (2019) 42:169–81. 10.1037/prj0000356 30843721

[29] Butcher I Berry K Haddock G. Understanding individuals’ subjective experiences of negative symptoms of schizophrenia: a qualitative study. *Br J Clin Psychol.* (2020) 59:319–34. 10.1111/bjc.12248 32242945

[30] Hurtado MM Villena-Jimena A Quemada C Morales-Asencio JM. ). ‘I do not know where it comes from, I am suspicious of some childhood trauma’ association of trauma with psychosis according to the experience of those affected. *Eur J Psychotraumatol.* (2021) 12:1940759. 10.1080/20008198.2021.1940759 34367524 PMC8312611

[31] Wood L Williams CJ Billings J Johnson S. The therapeutic needs of psychiatric in-patients with psychosis: a qualitative exploration of patient and staff perspectives. *BJPsych Open.* (2019) 5:e45. 10.1192/bjo.2019.33 PMC6537445 31530314

[32] Vallath S Ravikanth L Regeer B Borba PC Henderson DC Scholte WF. Traumatic loss and psychosis – Reconceptualising the role of trauma in psychosis. *Eur J Psychotraumatol.* (2020) 11:1725322. 10.1080/20008198.2020.1725322 32341762 PMC7170325

[33] Campodonico C Varese F Berry K. Trauma and psychosis: a qualitative study exploring the perspectives of people with psychosis on the influence of traumatic experiences on psychotic symptoms and quality of life. *BMC Psychiatry.* (2022) 22:213. 10.1186/s12888-022-03808-3 35331194 PMC8944047

[34] Hartog I Scherer-Rath M Kruizinga R Netjes J Henriques J Nieuwkerk P Narrative meaning making and integration: toward a better understanding of the way falling ill influences quality of life. *J Health Psychol.* (2020) 25:738–54. 10.1177/1359105317731823 28948830 PMC7221864

[35] McAdams DP. Narrative identity. In: Schwartz SJ Luyckx K Vignoles VL editors. *Handbook of identity theory and research.* New York, NY: Springer New York (2011). p. 99–115.

[36] Habermas T. Autobiographical reasoning: arguing and narrating from a biographical perspective. *N Direct Child Adolesc Dev.* (2011) 2011:1–17. 10.1002/cd.285 21387528

[37] Dimaggio G Lysaker PH. Metacognition and mentalizing in the psychotherapy of patients with psychosis and personality disorders. *J Clin Psychol.* (2015) 71:117–24. 10.1002/jclp.22147 25557317

[38] McAdams DP. *The redemptive self: Stories Americans live by-revised and expanded edition.* Oxford: Oxford University Press (2013).

[39] American Psychological Association. *Clinical practice guideline for treatment of posttraumatic stress disorder (PTSD) in adults.* Washington, DC: American Psychiatric Association (2017).

[40] Kleber RJ. Trauma and public mental health: a focused review. *Front Psychiatry.* (2019) 10:451. 10.3389/fpsyt.2019.00451 31293461 PMC6603306

[41] Neimeyer RA. Narrative disruptions in the construction of the self. In: Neimeyer RA Raskin JD editors. *Constructions of disorder: Meaning-making frameworks for psychotherapy.* Washington, DC: American Psychological Association (2000). p. 207–42.

[42] Neimeyer RA Tschudi F. Community and coherence: narrative contributions to the psychology of conflict and loss. *Narrat Conscious.* (2003) 8:166–91.

[43] Neimeyer RA Herrero O Botella L. Chaos to coherence: psychotherapeutic integration of traumatic loss. *J Construct Psychol.* (2006) 19:127–45. 10.1080/10720530500508738

[44] Vassilieva J. *Narrative psychology: Identity, transformation and ethics.* London: Palgrave Macmillan (2016).

[45] Bruner J. The narrative construction of reality. *Crit Inquiry.* (1991) 18:1–21. 10.1086/448619

[46] Park CL. Making sense of the meaning literature: an integrative review of meaning making and its effects on adjustment to stressful life events. *Psychol Bull.* (2010) 136:257–301. 10.1037/a0018301 20192563

[47] Kauffman J. *Loss of the assumptive world: A theory of traumatic loss.* London: Routledge (2013).

[48] Adler JM Lodi-Smith J Philippe FL Houle I. The incremental validity of narrative identity in predicting well-being: a review of the field and recommendations for the future. *Pers Soc Psychol Rev.* (2016) 20:142–75. 10.1177/1088868315585068 25968138

[49] Vanaken L Bijttebier P Fivush R Hermans D. An investigation of the concurrent and longitudinal associations between narrative coherence and mental health mediated by social support. *J Exp Psychopathol.* (2022) 13:20438087211068215. 10.1177/20438087211068215

[50] Bourdeau G Lecomte T Lysaker PH. Stages of recovery in early psychosis: associations with symptoms, function, and narrative development. *Psychol Psychother.* (2015) 88:127–42. 10.1111/papt.12038 25139504

[51] Mazor Y Gelkopf M Mueser KT Roe D. Posttraumatic growth in psychosis. *Front Psychiatry.* (2016) 7:202. 10.3389/fpsyt.2016.00202 28066275 PMC5165025

[52] Jansen JE Pedersen MB Trauelsen AM Nielsen HG Haahr UH Simonsen E. The experience of childhood trauma and its influence on the course of illness in first-episode psychosis: a qualitative study. *J Nerv Mental Dis.* (2016) 204:210–6. 10.1097/NMD.0000000000000449 26675249

[53] Cowan HR Mittal VA McAdams DP. Narrative identity in the psychosis spectrum: a systematic review and developmental model. *Clin Psychol Rev.* (2021) 88:102067. 10.1016/j.cpr.2021.102067 34274799 PMC8376804

[54] Berna F Potheegadoo J Aouadi I Ricarte JJ Allé MC Coutelle R A meta-analysis of autobiographical memory studies in schizophrenia spectrum disorder. *Schizoph Bulletin* (2016) 42:56–66. 10.1093/schbul/sbv099 26209548 PMC4681554

[55] Roe D Davidson L. Self and narrative in schizophrenia: time to author a new story. *Med Hum.* (2005) 31:89–94. 10.1136/jmh.2005.000214 23674667

[56] McGlashan TH Levy ST Carpenter WT Jr. Integration and sealing over: clinically distinct recovery styles from schizophrenia. *Arch Gen Psychiatry.* (1975) 32:1269–72. 10.1001/archpsyc.1975.01760280067006 1180660

[57] De Jager A Rhodes P Beavan V Holmes D McCabe K Thomas N Investigating the lived experience of recovery in people who hear voices. *Qual Health Res.* (2016) 26:1409–23. 10.1177/1049732315581602 25896792

[58] McGlashan TH. Recovery style from mental illness and long-term outcome. *J Nerv Ment Dis.* (1987) 175:681–5. 10.1097/00005053-198711000-00006 3681279

[59] Thompson KN McGorry PD Harrigan SM. Recovery style and outcome in first-episode psychosis. *Schizoph Res.* (2003) 62:31–6. 10.1016/s0920-9964(02)00428-0 12765740

[60] Staring ABP van der Gaag M Mulder CL. Recovery style predicts remission at one-year follow-up in outpatients with schizophrenia spectrum disorders. *J Nerv Mental Dis.* (2011) 199:295–300. 10.1097/NMD.0b013e3182174e97 21543947

[61] Zizolfi D Poloni N Caselli I Ielmini M Lucca G Diurni M Resilience and recovery style: a retrospective study on associations among personal resources, symptoms, neurocognition, quality of life and psychosocial functioning in psychotic patients. *Psychol Res Behav Manage.* (2019) 12:385–95. 10.2147/PRBM.S205424 31213935 PMC6549482

[62] Adler JM Dunlop WL Fivush R Lilgendahl JP Lodi-Smith J McAdams DP Research methods for studying narrative identity: a primer. *Soc Psychol Pers Sci.* (2017) 8:519–27. 10.1177/1948550617698202

[63] Elliott J. *Using narrative in social research: Qualitative and quantitative approaches.* Thousand Oaks, CA: SAGE (2008).

[64] Ponterotto JG. Qualitative research in counseling psychology: a primer on research paradigms and philosophy of science. *J Counsel Psychol.* (2005) 52:126–36. 10.1037/0022-0167.52.2.126

[65] Spector-Mersel G Knaifel E. Narrative research on mental health recovery: two sister paradigms. *J. Mental Health.* (2018) 27:298–306. 10.1080/09638237.2017.1340607 28648112

[66] van Sambeek N Baart A Franssen G van Geelen S Scheepers F. Recovering context in psychiatry: what contextual analysis of service users’ narratives can teach about recovery support. *Front Psychiatry.* (2021) 12:773856. 10.3389/fpsyt.2021.773856 34987427 PMC8720875

[67] American Psychiatric Association. *Diagnostic and statistical manual of mental disorders.* Washington, DC: American Psychiatric Association. (2013).

[68] Krupnik V. Trauma or adversity? *Traumatology.* (2019) 25:256–61. 10.1037/trm0000169

[69] Buswell G Haime Z Lloyd-Evans B Billings J. A systematic review of PTSD to the experience of psychosis: prevalence and associated factors. *BMC Psychiatry.* (2021) 21:9. 10.1186/s12888-020-02999-x 33413179 PMC7789184

[70] Murray M Sools A. Narrative research in clinical and health psychology. In: Rohleder P Lyons AC editors. *Qualitative research in Clinical and Health Psychology.* London: Palgrave Macmillan (2015). p. 133–54.

[71] Spector-Mersel G. Narrative research: time for a paradigm. *Narrat Inquiry.* (2010) 20:204–24. 10.1075/ni.20.1.10spe 33486653

[72] Habermas T Bluck S. Getting a life: the emergence of the life story in adolescence. *Psychol Bull.* (2000) 126:748–69. 10.1037/0033-2909.126.5.748 10989622

[73] Adler JM Waters TEA Poh J Seitz S. The nature of narrative coherence: an empirical approach. *J Res Pers.* (2018) 74:30–4. 10.1016/j.jrp.2018.01.001

[74] O’Brien BC Harris IB Beckman TJ Reed DA Cook DA. Standards for reporting qualitative research: a synthesis of recommendations. *Acad Med.* (2014) 89:1245–51. 10.1097/ACM.0000000000000388 24979285

[75] Myers NAL Ziv T. ‘No One Ever Even Asked Me that Before’: autobiographical power, social defeat, and recovery among African Americans with lived experiences of psychosis. *Med Anthropol Q.* (2016) 30:395–413. 10.1111/maq.12288 26990015

[76] Amsalem D Hasson-Ohayon I Gothelf D Roe D. Subtle ways of stigmatization among professionals: the subjective experience of consumers and their family members. *Psychiatr Rehabil J.* (2018) 41:163–8. 10.1037/prj0000310 29985015

[77] Klaver K Baart A. Attentiveness in care: towards a theoretical framework. *Nurs Ethics.* (2011) 18:686–93. 10.1177/0969733011408052 21788284

[78] Luhrmann TM Marrow J. *Our most troubling madness: Case studies in schizophrenia across cultures.* Berkeley, CA: University of California Press (2019).

[79] Thornhill H Clare L May R. Escape, enlightenment and endurance. *Anthropol Med.* (2004) 11:181–99. 10.1080/13648470410001678677 26868201

[80] Seikkula J Alakare B. Open dialogues with patients with psychosis and their families. In: Romme M Escher S editors. *Psychosis as a personal crisis*. London: Routledge (2013). p. 116–28.

[81] Hamm JA Beasley RE Mazor Y. Trauma and meaning-making in the recovery of the self: Implications for metacognitive reflection and insight therapy (MERIT). In: Hasson-Ohayon I Lysaker PH editors. *The recovery of the self in psychosis.* London: Routledge (2021). p. 164–81.

[82] Ermers NJ Franssen GEHI Scheepers FE van Sambeek N Geelen S. Illness insight in mental health care. In Diagnostic – conformity to co-narration of self-insight. (2023) (manuscript submitted for publication).

[83] Lysaker PH Roe D Yanos PT. Toward understanding the insight paradox: internalized stigma moderates the association between insight and social functioning, hope, and self-esteem among people with schizophrenia spectrum disorders. *Schizoph Bull.* (2007) 33:192–9. 10.1093/schbul/sbl016 16894025 PMC2012366

[84] Steel C. Hallucinations as a trauma-based memory: implications for psychological interventions. *Front Psychol.* (2015) 6:1262. 10.3389/fpsyg.2015.01262 26441698 PMC4569972

[85] Peach N Alvarez-Jimenez M Cropper SJ Sun P Halpin E O’Connell J Trauma and the content of hallucinations and post-traumatic intrusions in first-episode psychosis. *Psychol Psychother.* (2021) 94(Suppl 2):223–41. 10.1111/papt.12273 32154644

[86] van den Berg D Tolmeijer E Jongeneel A Staring ABP Palstra E van der Gaag M Voice phenomenology as a mirror of the past. *Psychol Med.* (2022) 53:2954–62. 34991770 10.1017/S0033291721004955 PMC10235665

[87] Meyer JM Farrell NR Kemp JJ Blakey SM Deacon BJ. Why do clinicians exclude anxious clients from exposure therapy? *Behav Res Therapy.* (2014) 54:49–53. 10.1016/j.brat.2014.01.004 24530499

[88] van den Berg DPG van der Vleugel BM de Bont PAJM Thijssen G de Roos C de Kleine R Exposing therapists to trauma-focused treatment in psychosis: effects on credibility, expected burden, and harm expectancies. *Eur J Psychotraumatol.* (2016) 7:31712. 10.3402/ejpt.v7.31712 27606710 PMC5015638

[89] Chadwick E Billings J. Barriers to delivering trauma-focused interventions for people with psychosis and post-traumatic stress disorder: a qualitative study of health care professionals’ views. *Psychol Psychother.* (2022) 95:541–60. 10.1111/papt.12387 35124894 PMC9304310

[90] Boevink WA. From being a disorder to dealing with life: an experiential exploration of the association between trauma and psychosis. *Schizoph Bull.* (2006) 32:17–9. 10.1093/schbul/sbi068 16166604 PMC2632178

[91] Hampson M Watt B Hicks R. Understanding the recovery process in psychosis. *J Recov Mental Health.* (2019) 2:35–44.

[92] de Bont PAJM van den Berg DPG van der Vleugel BM de Roos C de Jongh A van der Gaag M Prolonged exposure and EMDR for PTSD v. a PTSD waiting-list condition: effects on symptoms of psychosis, depression and social functioning in patients with chronic psychotic disorders. *Psychol Med.* (2016) 46:2411–21. 10.1017/S0033291716001094 27297048

[93] Sin J Spain D. Psychological interventions for trauma in individuals who have psychosis: a systematic review and meta-analysis. *Psychosis.* (2017) 9:67–81. 10.1080/17522439.2016.1167946

[94] Swan S Keen N Reynolds N Onwumere J. Psychological interventions for post-traumatic stress symptoms in psychosis: a systematic review of outcomes. *Front Psychol.* (2017) 8:341. 10.3389/fpsyg.2017.00341 28352239 PMC5348513

[95] van den Berg D de Bont PAJM van der Vleugel BM de Roos C de Jongh A van Minnen A Long-term outcomes of trauma-focused treatment in psychosis. *Br J Psychiatry.* (2018) 212:180–2. 10.1192/bjp.2017.30 29436320

[96] Burger SR Hardy A van der Linden T van Zelst C de Bont PAJ van der Vleugel B The bumpy road of trauma-focused treatment: posttraumatic stress disorder symptom exacerbation in people with psychosis. *J Traum Stress.* (2023) 36:299–309. 10.1002/jts.22907 36719408

[97] Luhrmann TM. Social defeat and the culture of chronicity: or, why schizophrenia does so well over there and so badly here. *Cult Med Psychiatry.* (2007) 31:135–72. 10.1007/s11013-007-9049-z 17534703

[98] van Nierop M Van Os J Gunther N Van Zelst C De Graaf R Ten Have M Does social defeat mediate the association between childhood trauma and psychosis? Evidence from the NEMESIS-2 S Tudy. *Acta Psychiatr Scand.* (2014) 129:467–76. 10.1111/acps.12212 24571736

[99] Veling W Susser E. Migration and psychotic disorders. *Expert Rev Neurotherap.* (2011) 11:65–76. 10.1586/ern.10.91 21158556

[100] Plummer K. ‘Whose Side Are We On?’ revisited: narrative power, narrative inequality, and a politics of narrative humanity. *Symbol Interact.* (2020) 43:46–71. 10.1002/symb.449

[101] Llewellyn-Beardsley J Rennick-Egglestone S Pollock K Ali Y Watson E Franklin D ‘Maybe I Shouldn’t Talk’: the role of power in the telling of mental health recovery stories. *Qual Health Res.* (2022) 32:1828–42. 10.1177/10497323221118239 35979858 PMC9511241

