# Supplementary material for: Making meaning of trauma in psychosis
Source: Front Psychiatry. 2023 Nov 3;14:1272683. doi: 10.3389/fpsyt.2023.1272683 (PMC10656619; doi:10.3389/fpsyt.2023.1272683)
Supplement: Supplementary file 1 [file Table_1.DOCX]

Supplementary Material

Appendix I: Interview guide

| **Main story and meaning** | |
| --- | --- |
| Opening question: What story would you like to share with us?  Alternative question: Could you tell what motivated you to share your story? | |
| **Topics to inquire**  *(to inquire when not brought up spontaneously)* | **Example questions**  *(to be adjusted to evolving story)* |
| Identity | How did this story affect the way you perceive yourself?  How did it affect the way others perceive you? |
| Participation | How did this story affect your work/activities?  How did this story affect your position in society? |
| Connectedness | How did people react to your story ?  How was the support from your loved-ones to your story? |
| Care | What role did mental health care play in your story?  What helped or hindered in care?  Would you like to change anything in mental healthcare? |
| Meaning | How do you look back on what happened  How do you look at your future after what happened? |

Appendix II: interviewer evaluation

Name of the interviewer:

| Skills | Yes/no/sometimes | Explanations and examples |
| --- | --- | --- |
| Gives the interviewee time to express thoughts: does not fill silences or interrupt unnecessarily. |  |  |
| Inquires further on topics that seem relevant for the interviewee. |  |  |
| Questions are asked in such way that more depth is created. |  |  |
| Asks about concrete situations and examples. |  |  |
| Does not ask suggestive questions. |  |  |
| Poses one question at a time. |  |  |
| Summarizes in an appropriate way (only if needed and in the words of the interviewee) |  |  |
| Reacts supportive and emphatic to what is told. |  |  |
| Reacts without judgement. |  |  |
| Ensures that all interview topics are covered (if not addressed spontaneously) |  |  |
| Is capable of guarding boundaries. |  |  |

| What went well in this interview? |
| --- |
| What are the most important learning points? |

Appendix III: Storyline Analysis

Narrative analysis based on (and adapted to this study) from:

*Murray M, Sools A. Narrative research in clinical and health psychology. In: Rohleder P, Lyons AC, ed. Qualitative research in clinical and health psychology London: Palgrave Macmillan; 2015. p. 133-54.*

**I Introduction**

*Case introduction*

Narrator: demographic characteristics, diagnosis and care pathway *[factual/descriptive information based on intake information]*

Interview context: location, interviewer characteristics, participants’ motivation for participation, evaluation of the interview.

Summary of main story *[in respondents own words]*

- *If main story and trauma storyline coincide skip step 2*

**II Trauma storyline analysis: content**

- *If trauma storyline is too minimal, analyze main storyline and try to make sense of minimal trauma storyline.*

Wording/specification of traumatic experience: What exact words does the narrator use to refer to trauma; What kind of traumatic experiences does the narrator describe (e.g., sexual abuse, physical assault)?

Meaning-making of trauma and psychosis: How are psychosis and trauma related for the narrator?

Characterization of storyline: Where and how in story does this storyline arise?

Story elements and breach

Actor: Who? Characterization of protagonist, traits.

Action/Events: What happens? In doing so, distinguish what happens to the protagonist and what he or she does. *[Balance between action and events balance gives an indication of the degree of agency].*

Setting/scene: Where? This can be either a description of a physical or mental setting.

Purpose, intention: Why? This can be either something the protagonist wants to achieve or desires, or the avoidance of a feared outcome.

Means/helpers and hinderers: By what or whom are goals/intentions achieved?

Breach: Between which of the above story elements is the imbalance? To identify the breach ask: What tension motivates the narrative? Where is the disruption in meaning-making? Where does not occur what is expected? If applicable: also describe resolution of the breach.

Narrative summary: summarize the story, based on the above elements, using the respondent's own words, making clear the connection/causality from their perspective.

**III Trauma storyline analysis: form**

Characterization of disintegration (form): How detailed, complex and coherent is the storyline?

- *Use indicators of (des)integration from the narrative identity literature:*

Causal Coherence: the narrator connects trauma (past), to his/her current identity (present), and (im)possibilities/direction in life (future). Thus, continuity in experience of self before, during and after the traumatic experience is restored. The opposite pattern is for the narrator to describe trauma as external, foreign and separate from self. The person before the trauma and the person after it cannot be united (adaptation/reconstruction of the self-story has then not taken place sufficiently).

Thematic coherence: The narrative is centered around an overarching theme, life lesson, value or principle that is the result of reinterpretation and reflection. This reduces friction in meaning. The opposite is a story without a clear plot, in which individual events and experiences are described, but not connected and evaluated.

The storyline is detailed versus unelaborated. Detail involves clarity of various story elements (who, what, where, when) as well as thoughts and feelings associated with the trauma.

Recovery style: The person turns toward the traumatic experience and actively examines it (integration style). The opposite pattern as something to be moved away from (sealing over style) and to see the traumatic experience as external to the self.

*Use Neimeyer’s three forms of narrative disruption as sensitizing concepts to recognize and elaborate different forms of narrative disruption:*

Disintegration of self; the person before the trauma and after the trauma cannot be reconciled (requires adjustment of conceptions).

Narrative dissociation: the trauma narrative does not become part of the socially shared narrative (this is where repression and avoidance, among others, play a role).

Narrative dominance: one's own attempts to make meaning are dominated by a dominant narrative (e.g., stigmatized illness identity).

**IV Interactional narrative analysis**

Positioning of storylines: How are the storylines positioned in the interaction? Which ones seem obvious, which ones have dead ends, which ones are encouraged by the interviewer or not. Missing elements, 'gaps' and ambiguity are also important to clarify.

Positioning of narrator and listener: Who decides which storyline is desirable? And which ones are disqualified?

Conclusion about what is at stake: Interpretation from previous steps that tries to explain positioning.

**V Contextual analysis**

*Note: here the approach shifts from emic to etic. Theoretical knowledge of the researcher plays a larger role here and may differ from the narrator's own meaning-making.*

Positioning narratives in broader social, societal and political context: What views of the narrator's characteristics prevail in society? How does the story relate to dominant narratives? Where do norms and values of the speaker and the outside world clash? This is also about values/moral context to which the narrator and narrative relate.

**VI Conclusions**

What breach is central to this self-story and what is the meaning of trauma in it? (content of meaning made)

To what extent is the trauma storyline integrated into the self-story as a whole? (form of meaning-made)

What individual, interactional and social factors seem to play a role in (dis)integration in this story?

What barriers and facilitators in the integration of trauma can be identified? (process of meaning-making)

When meaning-making is the goal of the narrator, take self-identified helpers and hinderers at a higher level of abstraction: what appear to be the "working mechanisms”?

When meaning-making is not the narrator's goal, interpret why this might be so from previous conclusions.

**VII comparative analysis (after analyzing all stories).**

Look across all cases for similarities and differences at the level of a) plot/breach; b) meaning of trauma within self-story c) characteristics of (lack of)integration; d) barriers and facilitators in the meaning-making process.

**
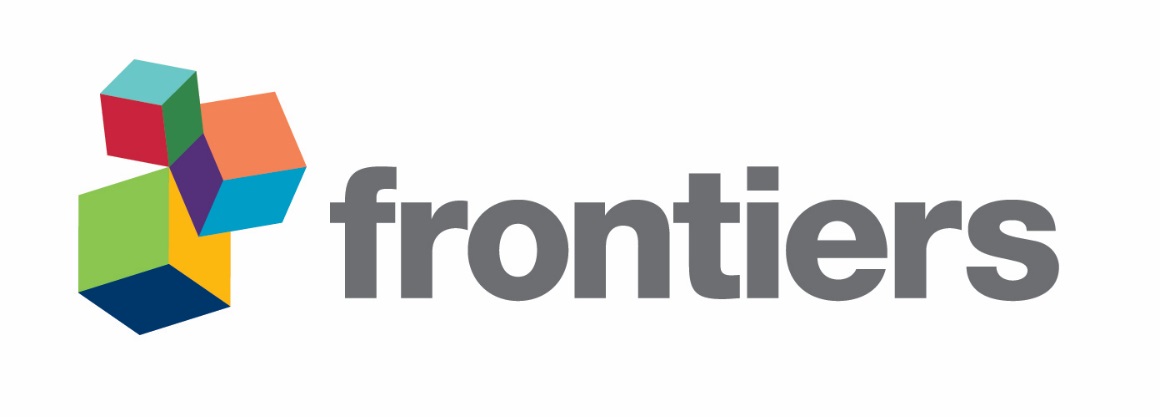
**
